# Changes in Metabolic Profile in the Women with a History of PCOS—A Long-Term Follow-Up Study

**DOI:** 10.3390/jcm9103367

**Published:** 2020-10-20

**Authors:** Małgorzata Jacewicz-Święcka, Irina Kowalska

**Affiliations:** 1 Department of Endocrinology, Diabetology and Internal Medicine, Medical University of Bialystok, 15-089 Białystok, Poland; 2 Department of Internal Medicine and Metabolic Diseases, Medical University of Bialystok, 15-089 Białystok, Poland; irinak@poczta.onet.pl

**Keywords:** PCOS, prediabetes, insulin resistance, metabolic syndrome, longitudinal study

## Abstract

Data concerning metabolic consequences in women with polycystic ovary syndrome (PCOS) are delivered mainly by cross-sectional studies. In this research, we re-examined 31 Caucasian PCOS women after a median period of 120.9 months to evaluate the changes in metabolic syndrome components. Clinical examination, oral glucose tolerance test with estimations of glucose and insulin, lipids, sex hormone-binding globulin (SHBG) and sex hormones assessments were performed on two occasions. Additionally, the euglycaemic hyperinsulinaemic clamp technique was used at the baseline to assess insulin sensitivity (M-clamp value). In the end, the median age of participants was 35. We observed an increase in glucose concentrations, a decrease in insulin concentrations and no changes in insulin resistance markers. Final mean glucose, mean insulin, Matsuda index and body mass index (BMI) were correlated with baseline M-clamp value and SHBG (*p* < 0.01). During the follow-up, no one in the sample developed diabetes. The annualised incidence rate for conversion from normoglycaemia to prediabetes totalled 4.5%. Baseline BMI, free androgen index, fasting glucose and M-clamp value were identified as prediabetes predictors in young PCOS women (respectively, OR = 1.17, OR = 1.42, OR = 1.2, OR = 0.73, *p* < 0.05). Prediabetes appeared in 76.47% of the women with a final BMI of ≥ 25 kg/m^2^ and in 7.14% of the normal-weight women (*p* = 0.0001). In conclusion, we report a high rate of adverse change in glucose metabolism in overweight and obese participants, a deterioration in β-cell function and strong correlations between metabolic parameters assessed in the third and the fourth decade in PCOS women, emphasising the role of early intervention to prevent cardiometabolic diseases.

## 1. Introduction

Polycystic ovary syndrome (PCOS) is the most frequent endocrine disorder among women of reproductive age with various prevalence and manifestation among populations [1,2,3]. In the last few years, different sets of diagnostic criteria were proposed and currently, the revised Rotterdam criteria are recommended [4].

The nature of PCOS is multifactorial and heterogeneous. Environmental factors such as prenatal androgen exposure, poor fetal growth, high carbohydrate consumption and acquired obesity interact with genetic origins and contribute to PCOS pathogenesis [5,6,7,8,9]. It is well established that insulin resistance (IR) and compensatory hyperinsulinaemia are central aetiological abnormalities in women with PCOS which lead to the overproduction of ovarian and adrenal androgens and an increase in androgen bioavailability through inhibition of sex hormone-binding globulin (SHBG) secretion [10]. The prevalence of IR in women with PCOS is estimated at 50–70% [11], or according to another source, even as high as 75% in lean and 95% in overweight women [12]. However, the proper assessment is difficult due to the fact that IR is assessed using different methods. Cassar et al. in a meta-analysis of 28 articles concerning insulin sensitivity estimated with the euglycaemic hyperinsulinaemic clamp found that IR is the intrinsic feature of PCOS and that insulin sensitivity is 27% lower in women with PCOS compared to controls, independently of body mass index (BMI) [13]. The importance of insulin resistance phenomenon regardless of body weight was provided by several authors [14,15].

IR and hyperandrogenism (HA) along with insulin secretory defects, post-receptor defect in insulin signalling, low-grade inflammation, high carbohydrate consumption, hypothalamic–pituitary axis abnormalities and disrupted folliculogenesis contribute to the reproductive and metabolic dysfunctions in PCOS [5,10,16,17,18]. It has been described that obesity is threefold more prevalent in women with PCOS compared to healthy women [19]. Obesity synergistically with PCOS increases IR and hyperinsulinaemia [20,21] and affects worse metabolic prognosis [21,22]. The development of glucose abnormalities is a multifactorial process that can develop in PCOS subjects from adolescence [23] and may be exacerbated by ageing [24]. It has been found that even non-obese and young women with PCOS have a higher risk of prediabetes (preDM), diabetes mellitus type 2 (T2DM) and gestational diabetes mellitus compared to age- and weight-matched women without PCOS [25,26]. The prevalence of impaired glucose tolerance (IGT) and T2DM in PCOS patients totals 23–35% and 4–10%, respectively [18]. Furthermore, although obesity affects lipid profile, PCOS seems to be a completely independent risk factor of dyslipidaemia [27] which affects 70% of patients [28]. Elevated triglycerides (TG) and decreased high-density lipoprotein cholesterol (HDL-c) seem to be the most common pattern in PCOS [29]. There is also evidence that arterial hypertension (AH) is significantly more prevalent in premenopausal women with PCOS compared to the general population [30,31]. Consequently, the prevalence of metabolic syndrome (MetS) is found to be at least threefold higher in women with PCOS compared to general population [32] and is estimated at 33% to 47% [18,33]. Interestingly, although PCOS patients have a proven increased prevalence of cardiovascular disease risk factors, the risk of cardiovascular morbidity and mortality is still equivocal [34,35]. To explain this phenomenon, longitudinal studies with PCOS women should be conducted. The knowledge of the pace, the nature of the metabolic changes and the interrelationships between them in women with PCOS could be of key importance for the explanation of dissonance between the expected and observed cardiovascular outcomes.

Although a lot of reports concerning metabolic consequences in PCOS have been published, almost all of them were designed as cross-sectional studies. There is a paucity of studies with long-term follow-up in which the detailed metabolic profile in women with PCOS was re-evaluated [35] and therefore, the course and development of metabolic disorders as well as the factors which impact on the subsequent complications in women with PCOS are poorly explored. The main aim of the present study was to determine the impact of ageing on glucose metabolism, insulin sensitivity, anthropometric parameters, lipid profile and values of blood pressure (BP) in a cohort of 31 women with previously diagnosed PCOS according to the Rotterdam criteria. Additionally, connections between anthropometric, metabolic and biochemical parameters assessed at an interval of ten years were investigated among subjects and risk factors for preDM in PCOS were identified. The data obtained would assist clinicians in the assessment and management of risk in women with PCOS.

## 2. Materials and Methods

### 2.1. Study Population

Ninety-one potentially eligible women diagnosed with PCOS in the Outpatient Endocrinology and Gynaecology Clinics in Białystok between 2003 and 2009 were identified from records to participate in the study. Only patients with correct results of an oral glucose tolerance test (OGTT) at baseline were taken under consideration in the follow-up study. Unfortunately, no contact was found with thirty-four subjects because of the invalid address, phone number and surname. Fifty-seven women were contacted by phone for re-evaluation and twenty-six of them were excluded, because they refused participation in the study due to living too far away from Białystok (*n* = 19), they were pregnant or within 12 months of delivery (*n* = 3), in case of malnutrition (*n* = 1), chronic or acute infection (within the previous 30 days) (*n* = 1), history of neoplasm or other serious medical problem (*n* = 2). Finally, thirty-one Caucasian women were included in the analysis. The median age at the baseline was 25.53 years (interquartile range (IQr) = 21.51 − 29.22). The studied cohort (*n* = 31) and the women who did not participate in the follow-up examination (*n* = 60) did not differ statistically significantly with regard to age, BMI and waist circumference (all *p* > 0.05).

### 2.2. Baseline Study

When first examined, the diagnosis of PCOS was made according to the Rotterdam criteria [4]—minimum two out of three criteria were fulfilled: oligomenorrhoea/amenorrhea (OM/AM), polycystic ovarian morphology (PCOM) in ultrasound examination, hirsutism (>8 according to modified Ferriman–Gallwey score (mFGS)) or increased testosterone (TT)/free androgen index (FAI), after exclusion of related disorders. The recruitment, clinical examination, anthropometric measurements, bioelectric impedance analysis, an OGTT, lipids and sex hormone assessments were performed as described previously [36]. Median values of sex hormones totalled: TT = 0.63 ng/mL (IQr = 0.46 − 0.88), FAI = 4.63 (IQr = 3.67 − 10.3), luteinizing hormone = 7.8 mIU/mL (IQr = 4.9 − 10.8). A total of 22 women (71%) presented classic phenotype A (PCOM + OM + HA), 7 women presented phenotype D (PCOM + OM), one person presented phenotype B (OM + HA) and one person presented phenotype C (PCOM + HA). Insulin sensitivity was measured with a euglycaemic hyperinsulinaemic clamp technique according to DeFronzo et al. [37]. The rate of whole-body glucose uptake was calculated as the mean glucose infusion rate from 80 to 120 min, corrected for glucose space and normalised per kilogram of fat-free mass (M-clamp value) [38].

### 2.3. Ethical Approval

Before conducting the study, all participants were provided informed and written consent after full explanation of the purpose and nature of all procedures used. The study protocol was approved by the Ethics Committee of the Medical University of Białystok and followed the principles of the Declaration of Helsinki (reference no. R-I-002/347/2015; date of approval 24.09.2015).

### 2.4. Follow-Up Study

Between December 2015 and May 2017 thirty-one women were re-evaluated and baseline tests were repeated.

#### 2.4.1. Protocol of the Study

The comprehensive questionnaire, physical examination, anthropometric measurements, transvaginal ultrasound scans, bioelectrical impedance analysis, blood collection for biochemical tests, an OGTT were all performed on the same day in each woman, 3–5 days after spontaneous menstrual bleeding or at random in the presence of AM (after excluding pregnancy using appropriate test). All the participants declared that they had not received treatment with oral contraceptives, antiandrogens, insulin sensitizers or any other medication known to affect sex hormones or carbohydrate metabolism for at least three months before an examination. No woman had a diagnosis of preDM or T2DM before being submitted to an OGTT.

#### 2.4.2. Clinical Evaluation and Anthropometric Measurements

Thirty-one women completed a clinic visit during which anamnesis and physical examination were conducted by the same physician in all the participants. Detailed interview according to the special questionnaire was collected and data about menstruation, reproductive history, current and previous diseases, smoking, medication use, family history of T2DM, cardio-vascular diseases and neoplasm (≥1 s degree relative) were obtained.

Body height was measured with a Harpenden Stadiometer (Tanita, Tokyo, Japan) without shoes to 0.1 cm and body mass was measured with underwear on an electronic scale to 0.1 kg. BMI was calculated as the ratio of body mass in kilograms (kg) and height in meters squared (m^2^). Waist circumference (WC) was measured with subjects standing, using a 1-cm-wide outstretched measuring tape, to the nearest 0.0 or 0.5 cm midway between the lowest rib margin and an iliac crest [39]. The waist-to-hip ratio (WHR) was calculated. Arterial BP was measured with an electronic sphygmomanometer after women were sitting for at least 15 min and recorded data were the mean of two consecutive measurements performed at a 15-min interval. The presence of hirsutism, acne and androgenic alopecia was verified.

#### 2.4.3. Image Tests

Ovary ultrasound scans were performed for all the women (Voluson 730 Expert, GE Healthcare, Zipf, Austria). Fat mass (%) was assessed by multi-frequency bioelectrical impedance analysis using the InBody 770 Body Composition Analyzer (Biospace, Beverly Hills, CA, USA) in all participants.

#### 2.4.4. Biochemical Analyses

Blood samples were drawn after an overnight fast between 08.00 and 09.00 am and were either analysed immediately or stored at −80°C until analysed. A standardised 75-g OGTT was performed. Blood samples for measurements of serum insulin and plasma glucose were taken at baseline, 30, 60, 120 min after glucose ingestion. Plasma glucose concentrations were assessed by the hexokinase method. Serum insulin concentrations were assessed with the immunoradiometric method (DIAsource ImmunoAssays S.A., Belgium) (minimum detectable concentration (MDC): 1 µIU/mL; intra-assay coefficient of variation (CV)—below 2.2%, inter-assay CV—below 6.5%). There is no cross-reaction between human and animal proinsulins in this method. Fasting blood samples were used to assay glycated haemoglobin (HbA1c), SHBG, lipid and hormonal profile. HbA1c level was measured using high-performance liquid chromatography (Bio Rad, Hercules, California, United States). Plasma lipid concentrations (total cholesterol (TC), HDL-c, TG) were measured by enzymatic colorimetric method (Cobas c111, Roche Diagnostic Ltd., Switzerland). SHBG concentration was assessed with immunoradiometric method (DIAsource ImmunoAssays S.A., Angleur, Belgium) (intra-assay and inter-assay CV for SHBG: 5.2% and 5.8%). Serum concentrations of TT, dehydroepiandrosterone sulfate (DHEAS), androstenedione (A4) and 17-hydroxprogesterone (17-OHP) were measured with radioimmunoassay (DIAsource ImmunoAssays S.A., Belgium) (MDC for TT: 0.05 ng/mL, for DHEAS: 1.23 µg/dL, for A4: 0.03 ng/mL, for 17-OHP: 0.03 ng/mL; intra-assay and inter-assay CV for TT: 3.3% and 4.8%, for DHEAS: 3.6% and 6.5%, for A4: 3.2% and 5.9%, for 17-OHP: 6.8% and 10.7%).

#### 2.4.5. Calculations

IR was estimated using the homeostasis model assessment (HOMA) according to the following formula: (fasting serum insulin (µIU/mL)  ×  fasting plasma glucose (mmol/L))/22.5 [40], with a threshold of at least 2.5. Matsuda index was calculated using the validated calculator [41] and the values ≤ 2.5 indicated on IR. HOMA β-cell function (HOMA-%β) was evaluated according to the formula: (20  ×  fasting insulin (µIU/mL))/(fasting plasma glucose (mmol/L)—3.5) [40]. β-cell function was assessed also with the insulinogenic index (IGI) and calculated as (30 min insulin (µIU/mL)—fasting insulin (µIU/mL))/(30 min glucose (mg/dL)—fasting glucose (mg/dL)). FAI was calculated as serum (TT (nmol/L)  ×  100)/SHBG (nmol/L) [42]. Plasma low-density lipoprotein cholesterol (LDL-c) was calculated according to Friedewald’s formula.

#### 2.4.6. Statistical Analysis

All analysed variables were tested for normality of distribution using the Lilliefors (Kolomorov–Smirnov) and Shapiro–Wilk tests. Due to the non-normal distribution of the data, all values were expressed as median (interquartile range). Comparisons between the groups were evaluated with non-parametric Mann–Whitney U-test. Wilcoxon signed-rank tests and the sign test were used to compare estimated variables at baseline and after follow-up. Correlation analysis was performed using the Spearman test. Chi-square test was used to determine the between-group differences of categorical variables. Afterwards, logistic regression methods were used to assess which factors determined at baseline study predict prediabetes found at the end of a 10-year follow-up period. A *p*-value < 0.05 was considered statistically significant. The statistical analysis for the present study was performed with the Statistica package (Statistica 13.3, Statsoft, Cracow, Poland) and Stata/IC 12.1 (StataCorp LP).

#### 2.4.7. Definitions

PCOS was diagnosed as the presence of a minimum two out of the following conditions: clinical and/or biochemical HA, OM or AM, PCOM assessed on transvaginal ultrasound [4,43]. Clinical HA was assessed on the basis of hirsutism (mFGS > 8), acne or androgenic alopecia. Biochemical HA was defined as TT ≥ 70 ng/dL and/or FAI ≥ 5 and/or A4 > 3 ng/mL, and/or serum DHEAS above the level recommended for the respective age group. OM was defined as menstrual cycle intervals ≥ 35 days or < 8 cycles per year, while AM as menstrual cycle length > 90 days. Related disorders like thyroid dysfunction, hyperprolactinaemia, ovary tumours and non-classic congenital adrenal hyperplasia were excluded by appropriate testing.

Women were categorized into BMI groups for normal weight (18.5–24.9 kg/m^2^), overweight (25.0–29.9 kg/m^2^) and obese (BMI ≥ 30.0 kg/m^2^) [44]. AH was defined as mean systolic BP (SBP) ≥ 140 mmHg or mean diastolic BP (DBP) ≥ 90 mmHg or the use of antihypertensive medications. PreDM referred to impaired fasting glucose (IFG) when the fasting plasma glucose levels were 100–125 mg/dL and IGT when the 2-h plasma glucose values in an OGTT were 140–199 mg/dL. T2DM was defined as fasting plasma glucose ≥ 126 mg/dL, or the 2-h plasma glucose ≥ 200 mg/dL [45]. Dyslipidaemia includes hypercholesterolaemia (LDL-c ≥ 115 mg/dL or non-HDL-c > 145 mg/dL), hypertriglyceridaemia (TG ≥ 150 mg/dL), low concentration of HDL-c (<50 mg/dL) or use of cholesterol-lowering medications. MetS was defined according to the International Diabetes Federation [46]. The diagnosis of MetS necessarily requires elevated WC (≥80 cm) and positivity in ≥2 of 4 criteria, namely: elevated BP (SBP ≥ 130 mmHg or DBP ≥ 85 mmHg or antihypertensive drug treatment), reduced HDL-c (<50 mg/dL or the use of cholesterol-lowering drugs), elevated TG (≥150 mg/dL or specific drug treatment), and elevated blood glucose (IFG, IGT, T2DM).

## 3. Results

The median duration of the follow-up period was 120.9 months (IQr = 107.17 − 127.38). The median age which was reached at the end of the observation was 35 years (IQr = 31.2 − 39.8).

### 3.1. Anthropometric Parameters

During the observation median values of BMI and WC increased significantly in the entire group (Table 1).

It was noted that 94.12% of the women who in the past had a BMI of ≥ 25 kg/m^2^ remained overweight or obese, and similarly 92.86% of the women with BMI < 25 kg/m^2^ in the past remained normal weight after the follow-up period (*p* < 0.0001). However, 50% of the women with WC < 80 cm at the baseline finally developed abdominal obesity, while no woman with WC ≥ 80 cm normalised this parameter over time (*p* < 0.05). The characteristics of the women according to their final BMI are presented in Table 2.

Correlations were found between the final values of anthropometric parameters and baseline metabolic parameters (Table 3). Moreover, it is worth mentioning connections between anthropometric parameters and androgens assessed at the final examination. It was noted that BMI, WC and fat mass (%) were correlated with FAI (respectively, R = 0.38, R = 0.4, R = 0.4, all *p* < 0.05), and BMI was correlated with 17-OHP (R = −0.36, *p* < 0.05). A positive correlation was found between ∆BMI and ∆FAI, whereas a negative correlation was found between ∆BMI and ∆SHBG (respectively, R = 0.39, R = −0.40, all *p* < 0.05).

### 3.2. Blood Pressure

Median values of SBP and DBP did not change significantly in the entire group, however, three women developed AH and were taking antihypertensive medicines. Significantly higher values of SBP and DBP were found in the women with BMI ≥ 25 kg/m^2^ compared to the normal weight participants (Table 2), in the women with Matsuda-IR compared to the women without IR (respectively, 132 vs. 122 mmHg, 81 vs. 76 mmHg, all *p* < 0.05), and in the women with OM compared to the women with regular menstrual cycles (130 vs. 121 mmHg, 79 vs. 76 mmHg, all *p* < 0.05).

BP values were correlated with final anthropometric parameters (BMI, WC, WHR, fat mass %), insulin concentrations and IR indices (all *p* < 0.05). WC and fat mass (%) were the only metabolic parameters assessed at the baseline that were significantly correlated with final values of SBP (respectively, R = 0.49, *p* = 0.006; R = 0.38, *p* = 0.03), while fat mass (%) was the only metabolic parameter assessed at the baseline that was correlated with final values of DBP (R = 0.42, *p* = 0.02).

### 3.3. Lipids

TC, LDL-c, HDL-c and TG concentrations, as well as the percentages of patients with hypertriglyceridaemia, hypercholesterolaemia or low concentration of HDL-c, did not change markedLy in the entire group (Table 1). Abnormal lipid profile (elevated LDL-c or TG or lowered HDL-c) was found in 69% of the women who had had abnormal lipid profile and in 22% of the women who had had normal lipid concentrations at the baseline (*p* = 0.009). Disturbed lipid profile was detected in 70.59% of the women with BMI ≥ 25 kg/m^2^ and in 7.14% of normal weight women (*p* = 0.0004). Interestingly, lowered concentration of HDL-c was noted only in overweight or obese women. Furthermore, abnormal lipid profile was found in no woman with WC < 80 cm and in 57% of the women with abdominal obesity (*p* = 0.005). TG, HDL-c and non-HDL-c determined at the end of the follow-up were correlated with final BMI (respectively, R = 0.78, R = −0.68, R = 0.71, all *p* < 0.0001), WC (respectively, R = 0.77, R = −0.63, R = 0.7, all *p* < 0.001), SHBG (respectively, R = −0.55, R = 0.57, R = −0.61, all *p* < 0.001) and FAI (respectively, R = 0.41, *p* = 0.02; R = −0.51, *p* = 0.003; R = 0.52, *p* = 0.003), but also with baseline BMI (respectively, R = 0.79, R = −0.63, R = 0.68, all *p* < 0.001) and WC (respectively, R = 0.82, R = −0.62, R = 0.68, all *p* < 0.001). Interestingly, a low concentration of HDL-c was found in 42.11% of the women who had had biochemical HA at the baseline and in no one who had had not biochemical HA in the past (*p* = 0.009). Furthermore, lipids were also correlated with glucose, insulin and markers of IR (data depicted in the chapter “OGTT and IR”).

### 3.4. Glycaemic Status and Insulin Resistance

#### 3.4.1. OGTT and IR

No one of the participants ever used antidiabetics except metformin, however, at the final examination, no one declared taking metformin in the last year. After the follow-up all the concentrations of glucose determined during an OGTT increased significantly in the entire group, while all the concentrations of insulin decreased, however, only fasting insulin, 30-min insulin and HOMA-%β changed significantly. Interestingly, there were no significant changes in IR indices, like HOMA-IR score and Matsuda index in the examined participants and the percentage of the women with a diagnosis of IR decreased slightly. Changes in glucose metabolism in the entire group are presented in Table 4.

∆Fasting insulin was correlated with the initial value of fasting insulin and similarly, ∆HOMA-%β was correlated with the value of HOMA-%β assessed at the baseline (respectively, R = −0.57, *p* = 0.0008; R = −0.88, *p* < 0.0001). All the important correlations between glucose, insulin and markers of IR assessed at the end of the follow-up and selected anthropometric, metabolic and hormonal parameters are presented in Table 5 and Table 6.

A significant relationship between IR and abnormal lipid profile was noted. Dyslipidaemia was found in 70% of the women who were diagnosed with IR according to Matsuda index and in 29% of the women who had not IR (*p* = 0.03), whereas a low concentration of HDL-c could be observed in 60% of the women with Matsuda-IR and in 9.52% of the women without IR (*p* = 0.003).

It is worth mentioning that a connection between IR and androgens was found. Matsuda-IR could be found in 63.64% of the women with an elevated value of FAI and only in 15% of the women with not elevated FAI (*p* = 0.006). Statistically significant correlations were found between final FAI and both initial and final Matsuda index (respectively, R = −0.53, *p* = 0.002; R = −0.45, *p* = 0.01). On the other hand, the women diagnosed with Matsuda-IR had significantly lower concentrations of adrenal androgen (17-OHP) compared to the women without IR (respectively, 1.08, IQr: 0.75–1.16 vs. 1.38, IQr: 0.98–1.59, *p* = 0.004). We found 17-OHP to be almost significantly correlated with Matsuda index and fasting insulin (respectively, R = 0.33, R = −0.33, all *p* = 0.07).

#### 3.4.2. Incident Prediabetes

After a median follow-up of 10 years, subsequent glucose load measurements revealed that 45% of the studied women developed preDM (*n* = 14). Of all the participants, 25.8% developed IFG (*n* = 8), whereas 19.35% developed IGT (*n* = 6). No one developed T2DM. Thus, the annualised incidence rate for conversion from normoglycaemic to preDM was 4.5%, to IFG—2.6% and to IGT—1.9%. Differences between women with normal and abnormal glucose tolerance are presented in Table 7.

Of the women affected with preDM, 57% were obese, whereas 7.14% were normal weight. PreDM appeared in 76.47% of the women with final BMI ≥ 25 kg/m^2^ and in 7.14% of the normal-weight women (*p* = 0.0001). On the other hand, preDM appeared in 70.59% of the women who had been overweight or obese and in 14.29% of the women who had been normal weight at the onset of the observation (*p* = 0.002), as also in 80% of the women who had had abdominal obesity (WC ≥ 80 cm) in the past and in 12.5% of the women who had not had abdominal obesity (*p* = 0.0002). Interestingly, all the women who developed preDM had WC ≥ 80 cm at the final examination. Another connection was found between preDM and previous insulin sensitivity. M-clamp value estimated as lower than median (<8.438 mg/kgffm/min) was found in 71% of the women with preDM and in 29% of the women who stayed normoglycaemic (*p* = 0.02). It was also noted that preDM appeared more often in the women who had been diagnosed with MetS at the onset of the observation compared to those who had not fulfilled such criteria (35.71% vs. 5.88%, *p* = 0.04).

Moreover, a connection was observed between HA noted at the beginning of the follow-up and later development of preDM. Almost 93% of the women with preDM de novo had been diagnosed with HA ten years earlier. On the other hand, preDM appeared in 63.16% of the women with previously elevated androgens and in 16.67% of the women without biochemical HA in the past (*p* = 0.01). The only significant difference in hormonal profile between the women with and without preDM concerned FAI.

Among 31 women with a previous diagnosis of PCOS the relationships between anthropometric, metabolic and hormonal parameters noted at the beginning of the observation and preDM found at the end of a 10-year follow-up period were assessed. The results show that BMI, FAI, fasting glucose and M-clamp value measured at the beginning of the observation are the most important predictors of preDM in PCOS women. All statistically significant predictors of preDM determined in logistic regression are presented in Table 8. SHBG measured at the onset of the observation was a marginally non-significant covariate associated with preDM (OR = 0.96; 95% CI: 0.93 to 1.0025, *p* = 0.067).

## 4. Discussion

The main finding of our study is the observation that the anthropometric and metabolic parameters assessed in young women with PCOS and ten years after are significantly correlated. This demonstrates that without the early therapeutic intervention in the affected subjects the adverse course of the metabolic changes will be continuing.

During the follow-up, we observed a spontaneous increase in the glucose concentrations, a decrease in the insulin concentrations and no changes in the indices of IR. Similar results were obtained by several authors [47,48,49,50]. This confirms the hypothesis that insulin sensitivity in patients with PCOS remains unchanged, whereas β-cell function decreases over time, which might be a result of ageing and exhaustion of pancreatic cells compensating IR over the long term.

The results of our study confirm a high risk of abnormal glucose tolerance in overweight/obese women with diagnosed PCOS [51]. The recently published data from the multi-centre national population health examination survey (WOBASZ) revealed that the prevalence of IFG in the cohort of Polish women aged 20–34 years totals 4.3%, which is much lower than in our PCOS cohort (25.8%) [52]. Available data concerning the development of preDM in PCOS women are limited. Pesant et al. reported an 8.8% annual conversion rate to abnormal glucose tolerance [48], but the mean BMI of the participants was 33.8 kg/m^2^—markedly higher compared to our cohort. Furthermore, we determined that BMI, glucose and insulin concentrations, IR, FAI and levels of TG and HDL-c are important risk factors for abnormal glucose tolerance in women with PCOS and these results confirm the previous reports [48,53,54,55]. Recent research emphasises the association between the diet and clinical severity of PCOS and indicates the Mediterranean diet as a primary preventive and therapeutic tool in women with PCOS, weakening insulin resistance and eventually promoting improvements of reproductive life and endocrine outcomes [56].

We demonstrate strong correlations in women with PCOS between SHBG and components of MetS, like obesity, lipid profile, IR and preDM, and that is in line with the studies performed both in PCOS women and the general population [53,57]. It seems that the main role in the relationship between HA presented as FAI and metabolic complications is played by SBHG, not by TT. Not surprisingly, SHBG is proposed as a clinically useful marker of IR and metabolic status in PCOS [13]. We confirm that the measurement of SHBG may have a role in the early identification of individuals at a high risk of abnormal glucose metabolism. Although HA is associated with an adverse metabolic phenotype in PCOS patients, previous studies have shown that DHEAS may have positive effects on fat distribution and IR in women with PCOS [58,59,60]. In our study, the negative connections are presented between both 17-OHP and obesity and 17-OHP and IR. However, because of the lack of similar reports, the relationship between 17-OHP and adverse metabolic outcomes requires more research.

We report a high prevalence of obesity and overweight in the PCOS cohort (respectively, 29% and 26%) in comparison with the general population of young Polish women (respectively, 6% and 14%) [52], and a significant increase in BMI, WC and the percentage of women with obesity, during the follow-up. Available data concerning the changes in anthropometric parameters in PCOS women are inconsistent. Significant increase as well as no changes in WC and BMI have been described [47,50,61,62]. This discrepancy may be caused by the differences in ethnicity and treatment strategies applied during the follow-up [63]. Nevertheless, we noticed no changes in BP and lipid concentrations in the studied women and the same results were obtained by the authors of the studies with similar duration of the follow-up [50,64,65]. A significant increase in the level of LDL-c was described in the studies with an over 20-year observation [49,62].

There are a number of limitations to the present study. Obviously, the main limitation is the small cohort size, which is connected with two factors. The first one is the fact of the restrictive inclusion criteria for the study. The number of women with PCOS and conducted clamp study was limited. The second one is a relatively high drop-out rate (66%). However, a comparable percentage can be found in similar studies [66]. The women who participated in the first examination were in their twenties and during ten years of the follow-up, some of them changed their names, phone numbers and addresses. A sudden increase in labour migration at the beginning of the 21st century had an additional impact on the fact, that some of the examined women stayed abroad and their re-evaluation was not possible. Another potential limitation is the fact that the sample was recruited from an outpatient clinic setting. We are aware that this cohort may contain women with more severe disease compared to the entire community. Consecutive shortcomings of this analysis are lack of controls, lack of information about lifestyle and dietetic habits among the participants, and the fact that at the follow-up, we used surrogate markers for assessing IR (HOMA score and Matsuda index)—not a gold standard clamp method. Additionally, a period of three months of withdrawal of hormonal therapy could be potentially insufficient to ensure “neutral” metabolic state.

However, our study also has a number of strengths, including the longitudinal design, appropriately diagnosed PCOS in the studied cohort, detailed biochemical assessment and comparable endocrine status in participating subjects. Another strength of this research is the fact that the same qualified person measured all the anthropometric parameters and recorded clinical data. Furthermore, it is one of the few longitudinal studies in which data concerning insulin sensitivity determined with a clamp technique in the women with PCOS are used. To our knowledge, it is the first longitudinal study of women with PCOS in which body fat was assessed by bioelectric impedance analysis both at the beginning and at the end of the follow-up.

We acknowledge that at the end of the follow-up observation, the studied cohort is still young and thus we can discuss only the increased risk rather than actual metabolic and cardiovascular consequences in our PCOS group. Further observation of an extended cohort is warranted to assess the development of type 2 diabetes and cardiovascular complications.

In conclusion, the study gives more insight into the metabolic changes in women with PCOS. In patients with PCOS, β-cell function decreases with age, whereas insulin sensitivity remains unchanged. PCOS women are at a high risk of preDM. Obesity, IR, fasting glucose and HA expressed as FAI are the most important predictors of glucose metabolism deterioration after the years of PCOS diagnosis. Serum SHBG is associated with metabolic complications and long-term prognosis in women with PCOS. A relationship between 17-OHP and metabolic outcomes requires further research.

## Figures and Tables

**Table 1:** Characteristics of the participants at the beginning and at the end of follow-up (*n* = 31).

Characteristic	Baseline	Follow-Up	*p*-Value
Age (years)	25.53 (21.51–29.22)	35.00 (31.20–39.80)	<0.00001
Body mass (kg)	70.0 (58.0–90.0)	72.2 (59.7–92.6)	0.002
BMI (kg/m^2^)	25.61 (21.48–31.42)	26.60 (21.76–34.29)	0.001
Waist Circumference (cm)	79.0 (71.0–97.0)	90.0 (77.0–110.0)	<0.00001
Waist to Hip Ratio	0.81 (0.77–0.85)	0.89 (0.85–0.95)	0.00006
Fat Mass (kg)	24.15 (15.96–35.15)	24.30 (16.44–37.50)	0.04
Fat Mass (%)	34.0 (27.5–44.0)	32.7 (26.47–45.0)	NS
Free Fat Mass (kg)	45.15 (42.24–50.76)	46.10 (44.0–55.5)	0.002
Triglycerides (mg/dL)	82 (61–135)	71 (57–119)	NS
Total Cholesterol (mg/dL)	179 (158–208)	196 (180–214)	NS
LDL-c (mg/dL)	93 (78.2–134.2)	104 (98.4–113.6)	NS
HDL-c (mg/dL)	58.2 (51–69)	68.0 (48–79)	0.07
Systolic Blood Pressure (mmHg)	120 (110–125)	124 (111–130)	0.08
Diastolic Blood Pressure (mmHg)	80 (70–80)	78 (72–82)	NS
Normal Weight *n* (%)	14 (45%)	14 (45%)	NS
Overweight *n* (%)	8 (25.86%)	5 (16.129%)	NS
Obesity *n* (%)	9 (29%)	12 (38.709%)	NS
Abdominal Obesity (waist circumference ≥ 80 cm) *n* (%)	15 (48%)	23 (74%)	0.04
Hypertriglyceridaemia *n* (%)	5 (16.129%)	5 (16.129%)	NS
Hypercholesterolaemia *n* (%)	9 (29%)	7 (22.58%)	NS
Low HDL-cholesterol *n* (%)	7 (22.58%)	8 (25.8%)	NS
Metabolic syndrome *n* (%)	6 (19.35%)	11 (35.48%)	NS

Data are expressed as median (25–75% quartiles) or numbers (%). *p* < 0.05 was considered statistically significant. Wilcoxon signed-rank test and the sign test were used to compare variables at baseline and after follow-up. BMI, body mass index; HDL-c, high-density lipoprotein cholesterol; LDL-c, low-density lipoprotein cholesterol; NS, non-significant.

**Table 2:** Metabolic characteristics of the participants according to BMI group.

Characteristic	BMI < 25 kg/m^2^ (*n* = 14)	BMI ≥ 25 kg/m^2^ (*n* = 17)	*p*-Value
Age at Follow-up (years)	34.15 (30.2–35.0)	38.3 (34.4–43.8)	0.01
BMI at Follow-up (kg/m^2^)	21.62 (20.32–22.87)	33.24 (28.83–39.12)	<0.00001
BMI at Baseline (kg/m^2^)	21.47 (19.66–21.94)	31.05 (27.82–36.29)	<0.00001
∆BMI (kg/m^2^)	0.67 (−0.71–1.18)	2.88 (1.14–4.04)	0.01
Waist Circumference at Follow-Up (cm)	77 (72–84)	109 (95–122)	<0.00001
Waist Circumference at Baseline (cm)	71 (68–74)	93 (85–103)	<0.00001
∆Waist Circumference (cm)	4.5 (0–10)	13 (8–19)	0.01
Waist To Hip Ratio at Follow-Up	0.84 (0.79–0.88)	0.93 (0.90–0.97)	0.0001
Waist To Hip Ratio at Baseline	0.78 (0.74–0.81)	0.85 (0.80–0.88)	0.006
Fat Mass At Follow-Up (%)	26.14 (22.1–30.5)	43.4 (37.5–48.4)	<0.00001
Fat Mass At Baseline (%)	27.1 (23.0–29.6)	42 (37.0–46.5)	<0.00001
Glucose 0’ at Follow-up (mg/dL)	89.5 (85–90)	102 (96–107)	0.00008
Glucose 0’ at Baseline (mg/dL)	81.5 (77–86.1)	86 (81–91)	0.06
Glucose 120’ at Follow-Up (mg/dL)	91.5 (77–99)	125 (100–152)	0.001
Glucose 120’ at Baseline (mg/dL)	72.5 (66–83.9)	100 (94–123)	0.006
Mean Glucose at Follow-Up (mg/dL)	100 (94–109.5)	147 (123.75–150.75)	0.00009
Mean Glucose at Baseline (mg/dL)	94.79 (86.75–104.5)	114.75 (102.25–125.75)	0.005
Insulin 0’ at Follow-Up (uIU/mL)	6.16 (5.09–8.20)	16.33 (11.54–19.57)	0.00002
Insulin 0’ at Baseline-Up (uIU/mL)	9.7 (7.07–13.94)	18.5 (11.70–25.34)	0.02
Insulin 120’ at Follow-Up (uIU/mL)	27.9 (18.09–31.82)	75.73 (33.57–149.26)	0.0009
Insulin 120’ at Baseline (uIU/mL)	27.56 (14.58–53.75)	84.67 (42.82–117.60)	0.01
Mean Insulin at Follow-Up (uIU/mL)	35.59 (28.91–45.61)	88.04 (52.66–107.55)	0.00006
Mean Insulin at Baseline (uIU/mL)	44.31 (36.27–63.26)	94.33 (67.47–116.93)	0.0009
M-Clamp Value At Baseline (mg/kgffm/min)	10.78 (8.71–12.13)	5.83 (4.17–8.32)	0.0002
Matsuda Index at Follow-Up	6.27 (5.62–7.32)	1.99 (1.7–3.06)	<0.00001
Matsuda Index at Baseline	4.93 (4.02–7.15)	2.45 (1.79–3.4)	0.0007
HOMA-IR Score at Follow-Up	1.36 (1.08–1.77)	3.87 (2.71–5.07)	<0.00001
HOMA-IR Score at Baseline	2.15 (1.35–2.66)	3.98 (2.51–5.54)	0.006
HOMA-%Β at Follow-Up	86.13 (73.31–115.16)	137.04 (95.96–192.38)	0.009
HOMA-%Β at Baseline	212.98 (164.77–288)	254.01 (175.5–349.2)	NS
Triglycerides at Follow-Up (mg/dL)	57 (48–63)	113 (83–186)	0.00001
Triglycerides at Baseline (mg/dL)	69.5 (48.6–82)	118 (76–155)	0.01
LDL-c at Follow-Up (mg/dL)	99.4 (77.6–110)	112.4 (103.8–132.2)	0.006
LDL-c at Baseline (mg/dL)	92.9 (78.2–105.18)	99 (86–139.8)	NS
HDL-c at Follow-Up (mg/dL)	73.5 (69–100)	50 (43–66)	0.0007
HDL-c at Baseline (mg/dL)	67.1 (58.2–74)	53 (42–63)	0.002
Systolic Blood Pressure At Follow-Up (mmHg)	121 (110–124)	126 (117–135)	0.04
Systolic Blood Pressure At Baseline (mmHg)	120 (110–120)	125 (110–130)	NS
Diastolic Blood Pressure At Follow-Up (mmHg)	75 (65–78)	79 (76–84)	0.03
Diastolic Blood Pressure At Baseline (mmHg)	72.5 (70–80)	80 (70–80)	NS
SHBG at Follow-up (nmol/l)	56.71 (44.79–105.09)	35.54 (23.36–66.32)	0.02
SHBG at Baseline (nmol/l)	60.85 (45.13–64.47)	27.20 (20.03–38.88)	0.0009
FAI at Follow-Up	2.20 (1.36–5.98)	2.80 (2.20–9.50)	NS
FAI at Baseline	4.11 (3.44–5.24)	8.83 (4.05–10.73)	0.04
17-OHP at Follow-Up (ng/mL)	1.52 (1.25–1.72)	1.05 (0.75–1.24)	0.008

Data are expressed as median (25–75% quartiles). *p* < 0.05 was considered statistically significant. *p*-value refers to differences between the two BMI groups. Variables were analysed by Mann–Whitney U-test. BMI, body mass index; FAI, free androgen index; HDL-c, high-density lipoprotein cholesterol; HOMA-IR, homeostasis model assessment of insulin resistance; HOMA-%β, homeostasis model assessment of β-cell dysfunction; LDL-c, low-density lipoprotein cholesterol; M-clamp value, insulin sensitivity estimated with the euglycaemic hyperinsulinaemic clamp technique; NS, non-significant; SHBG, sex hormone-binding globulin; 17-OHP, 17-hydroxyprogesterone.

**Table 3:** Correlations between final and baseline anthropometric and metabolic parameters.

Parameters at the Baseline Examination	Parameters at the Follow-Up		
	BMI (kg/m^2^)	Waist Circumference (cm)	Body Fat (%)
BMI (kg/m^2^)	0.94 ***	0.88 ***	0.85 ***
Waist Circumference (cm)	0.88 ***	0.88 ***	0.82 ***
Fat Mass (%)	0.89 ***	0.85 ***	0.87 ***
Glucose 0’ (mg/dL)	0.35 *^p^* ^= 0.05^	0.32 *^p^* ^= 0.08^	0.40 *
Mean Glucose (mg/dL)	0.42 *	0.45 *	0.51 **
Insulin 0’ (uIU/mL)	0.51 **	0.51 **	0.60 ***
Mean Insulin (uIU/mL)	0.61 ***	0.58 ***	0.72 ***
M-Clamp Value (mg/kgffm/min)	−0.68 ***	−0.65 ***	−0.70 ***
Matsuda Index	−0.62 ***	−0.61 ***	−0.75 ***
HOMA-IR Score	0.59 ***	0.59 ***	0.66 ***
Triglycerides (mg/dL)	0.55 **	0.46 **	0.56 ***
HDL-c (mg/dL)	−0.55 **	−0.49 **	−0.53 **
SHBG (nmol/l)	−0.52 **	−0.43 *	−0.56 **

Data are shown as r correlation coefficient. Significance levels are indicated by asterisks (*, *p* < 0.05; **, *p* < 0.01; ***, *p* < 0.001). Correlation coefficients were estimated by Spearman’s correlation for all parameters. BMI, body mass index; HDL-c, high-density lipoprotein cholesterol; HOMA-IR, homeostasis model assessment of insulin resistance; M-clamp value, insulin sensitivity estimated with the euglycaemic hyperinsulinaemic clamp technique; SHBG, sex hormone-binding globulin.

**Table 4:** Differences in glucose metabolism variables between baseline and follow-up investigations (*n* = 31).

Characteristic	Baseline	Follow-Up	*p*-Value
Glucose 0’ (mg/dL)	85 (80–89)	94 (89–103)	<0.00001
Glucose 30’ (mg/dL)	131 (123–152)	144 (125–169)	0.02
Glucose 60’ (mg/dL)	108 (91.6–137)	139 (97–166)	0.003
Glucose 120’ (mg/dL)	89 (70.5–102)	100 (88–131)	0.0006
Mean Glucose (mg/dL)	103.25 (91.58–122)	121.00 (99.75–147.25)	0.0005
Matsuda Index	3.64 (1.81–4.97)	3.95 (1.83–5.98)	NS
HOMA-IR score	2.65 (1.65–4.57)	2.12 (1.37–4.29)	NS
HOMA-%β	222.56 (169.70–346.50)	109.31 (82.33–167.74)	<0.00001
Insulinogenic Index	1.81 (1.10–2.65)	1.15 (0.78–2.2)	0.02
Insulin 0’ (µIU/mL)	12.51 (8.66–20.80)	8.64 (6.18–16.75)	0.003
Insulin 30’ (µIU/mL)	100 (68.16–126.37)	70.09 (50.37–111.3)	0.006
Insulin 60’ (µIU/mL)	94 (55.14–159.2)	88.33 (42.41–118.4)	NS
Insulin 120’ (µIU/mL)	48.8 (22.76–103.60)	33.57 (25.82–94.52)	NS
Mean Insulin (µIU/mL)	64.7 (38.75–102.15)	49.75 (35.62–90.72)	NS
Matsuda-IR *n* (%)	11 (35.4%)	10 (32.2%)	NS
HOMA-IR *n* (%)	17 (54.8%)	13 (41.9%)	NS

Data are expressed as median (25–75% quartiles) or numbers (%). *p* < 0.05 was considered statistically significant. Wilcoxon signed-rank test and the sign test were used to compare estimated variables at baseline and after follow-up. HOMA-IR, homeostasis model assessment of insulin resistance; HOMA-%β, homeostasis model assessment of β-cell dysfunction; NS, non-significant.

**Table 5:** Correlations between final concentrations of glucose, markers of insulin resistance and hyperinsulinaemia, with selected anthropometric, metabolic and hormonal parameters.

Selected Baseline and Final Metabolic and Hormonal Parameters	Final Glucose and Markers of Insulin Resistance					
	Glucose 0’ (mg/dL)	Glucose 120’ (mg/dL)	Mean Glucose (mg/dL)	Matsuda Index	HOMA-IR Score	HOMA-%β
Glucose 0’ at Baseline (mg/dL)	0.47 **	NS	NS	NS	0.39 *	NS
Glucose 120’ at Baseline (mg/dL)	0.45 *	0.52 **	0.49 **	−0.40 *	0.37 *	NS
Mean glucose at Baseline (mg/dL)	0.48 **	0.49 **	0.54 **	−0.41 *	0.38 *	NS
Matsuda Index at Baseline	−0.56 **	−0.53 **	−0.59 ***	0.75 ***	−0.71 ***	−0.63 ***
HOMA-IR Score at Baseline	0.49 **	0.36 *	0.43 *	−0.67 ***	0.69 ***	0.48 **
HOMA-%β at Baseline	NS	NS	NS	−0.36 *	NS	0.41 *
M-clamp Value at Baseline (mg/kgffm/min)	−0.41 *	−0.66 ***	−0.64 ***	0.71 ***	−0.68 ***	−0.63 ***
BMI at Follow-Up (kg/m^2^)	0.67 ***	0.48 **	0.60 ***	−0.80 ***	0.85 ***	0.57 ***
BMI at Baseline (kg/m^2^)	0.53 **	0.41 *	0.51 **	−0.73 ***	0.81 ***	0.62 ***
Waist Circumference at Follow-Up (cm)	0.66 ***	0.50 **	0.62 ***	−0.82 ***	0.84 ***	0.59 ***
Waist Circumference at Baseline (cm)	0.62 ***	0.48 **	0.66 ***	−0.83 ***	0.88 ***	0.61 ***
Fat Mass at Follow-Up (%)	0.67 ***	0.55 **	0.67 ***	−0.79 ***	0.82 ***	0.54 **
Fat mass at Baseline (%)	0.62 ***	0.48 **	0.63 ***	−0.73 ***	0.78 ***	0.50 **
Triglycerides at Follow-Up (mg/dL)	0.68 ***	0.52 ^p = 0.06^	0.67 ***	−0.79 ***	0.83 ***	0.52 **
Triglycerides at Baseline (mg/dL)	0.40 *	0.33 ^p = 0.07^	0.43 *	−0.46 **	0.59 ***	0.43 *
HDL-c at Follow-Up (mg/dL)	−0.61 ***	−0.35 ^p = 0.06^	−0.39 *	0.55 **	−0.65 ***	NS
HDL-c at Baseline (mg/dL)	−0.48 **	−0.34 ^p = 0.06^	−0.35 ^p = 0.05^	0.50 **	−0.62 ***	NS
SHBG at Follow-Up (nmol/l)	−0.48 **	NS	NS	0.56 ***	−0.58 ***	−0.35 ^p = 0.06^
SHBG at Baseline (nmol/l)	−0.42 *	−0.44 *	−0.51 **	0.55 **	−0.60 ***	−0.44 *
FAI at Follow-Up	0.44 *	NS	NS	−0.45 *	0.47 **	NS
FAI at Baseline	0.39 *	0.52 **	0.56 **	NS	0.36 ^p = 0.07^	NS

Data are shown as r correlation coefficient. Significance levels are indicated by asterisks (*, *p* < 0.05; **, *p* < 0.01; ***, *p* < 0.001). Correlation coefficients were estimated by Spearman’s correlation for all parameters. BMI, body mass index; FAI, free androgen index; HDL-c, high-density lipoprotein cholesterol; HOMA-IR, homeostasis model assessment of insulin resistance; HOMA-%β, homeostasis model assessment of β-cell dysfunction; M-clamp value, insulin sensitivity estimated with the euglycaemic hyperinsulinaemic clamp technique; NS, non-significant; SHBG, sex hormone-binding globulin.

**Table 6:** Correlations between final concentrations of insulin and selected anthropometric, metabolic and hormonal parameters.

Selected Baseline and Final Metabolic and Hormonal Parameters	Final Insulin		
	Insulin 0’ (µIU/mL)	Insulin 120’ (µIU/mL)	Mean Insulin (µIU/mL)
Insulin 0’ at Baseline (µIU/mL)	0.62 ***	0.52 **	0.61 ***
Insulin 120’ at Baseline (µIU/mL)	0.46 **	0.60 ***	0.38 *
Mean insulin at Baseline (µIU/mL)	0.62 ***	0.62 ***	0.74 ***
M-clamp Value at Baseline (mg/kgffm/min)	−0.71 ***	−0.67 ***	−0.67 ***
BMI at Follow-Up (kg/m^2^)	0.83 ***	0.52 **	0.73 ***
BMI at Baseline (kg/m^2^)	0.80 ***	0.45 *	0.66 ***
Waist circumference at Follow-Up (cm)	0.83 ***	0.57 ***	0.75 ***
Waist circumference at Baseline (cm)	0.87 ***	0.60 ***	0.74 ***
Fat mass at Follow-Up (%)	0.78 ***	0.61 ***	0.71 ***
Fat mass at Baseline (%)	0.74 ***	0.51 **	0.66 ***
Triglycerides at Follow-Up (mg/dL)	0.79 ***	0.62 ***	0.70 ***
Triglycerides at Baseline (mg/dL)	0.56 **	0.39 *	0.38 *
HDL-c at Follow-Up (mg/dL)	−0.56 **	−0.45 *	−0.52 **
HDL-c at Baseline (mg/dL)	−0.56 **	−0.39 *	−0.41 *
SHBG at Follow-Up (nmol/l)	−0.51 **	−0.50 **	−0.57 ***
SHBG at Baseline (nmol/l)	−0.57 ***	−0.46 **	−0.46 **
FAI at Follow-Up	0.42 *	0.46 **	0.46 **
FAI at Baseline	0.32 ^*p* = 0.08^	NS	NS

Data are shown as r correlation coefficient. Significance levels are indicated by asterisks (*, *p* < 0.05; **, *p* < 0.01; ***, *p* < 0.001). Correlation coefficients were estimated by Spearman’s correlation for all parameters. BMI, body mass index; FAI, free androgen index; HDL-c, high-density lipoprotein cholesterol; M-clamp value, insulin sensitivity estimated with the euglycaemic hyperinsulinaemic clamp technique; NS, non-significant; SHBG, sex hormone-binding globulin.

**Table 7:** Characteristics of the participants according to glucose metabolism.

Characteristic	Normal Glucose Tolerance (*n* = 17)	Prediabetes (*n* = 14)	*p*-Value
Age (years)	34.7 (30.2–37.3)	38.2 (34.4–43.8)	0.07
BMI at Follow-Up (kg/m^2^)	22.14 (20.83–24.8)	32.58 (27.13–39.12)	0.003
BMI at Baseline (kg/m^2^)	21.86 (20.59–25.78)	28.75 (25.61–36.29)	0.007
Waist circumference at Follow-Up (cm)	80 (75–90)	107 (94–122)	0.002
Waist circumference at Baseline (cm)	71 (68–78)	92.5 (80–103)	0.0007
Fat mass at Follow-Up (%)	27.7 (22.7–32.7)	44.2 (33.9–48.4)	0.002
Fat mass at Baseline (%)	28 (24–32)	41 (34.5–46.5)	0.002
Glucose 0′ at Follow-Up (mg/dL)	90 (87–91)	103 (101–108)	<0.0001
Glucose 0′ at Baseline (mg/dL)	80 (77–86)	87 (83–91.7)	0.006
Glucose 120′ at Follow-Up (mg/dL)	95 (77–98)	135 (118–159)	<0.0001
Glucose 120′ at Baseline (mg/dL)	74 (69–88)	101.5 (94–124)	0.003
Mean glucose at Follow-Up (mg/dL)	100.25 (95–109.5)	147.88 (134.75–152)	<0.0001
Mean glucose at Baseline (mg/dL)	98 (89.75–102.25)	120.46 (110.50–126.75)	0.002
Insulin 0′ at Follow-Up (uIU/mL)	6.83 (5.52–8.45)	16.22 (9.72–18.49)	0.002
Insulin 0′ at Baseline (uIU/mL)	10.53 (7.4–14.54)	16.18 (11.7–24.48)	NS
Insulin 120′ at Follow-Up (uIU/mL)	26.85 (18.09–31.82)	85.12 (45.86–149.26)	0.0002
Insulin 120′ at Baseline (uIU/mL)	29.21 (22.76–63.59)	85.76 (42.82–120.06)	0.04
Mean insulin at Follow-Up (uIU/mL)	35.68 (33.24–49.75)	71.44 (52.66–105.37)	0.005
Mean insulin at Baseline (uIU/mL)	52.93 (37.55–64.7)	88.79 (66.63–111.23)	0.03
M-clamp value at Baseline (mg/kgffm/min)	9.61 (8.4–11.62)	5.68 (4.15–8.44)	0.01
Matsuda index at follow-up	5.78 (5.2–7.19)	2.07 (1.72–3.17)	0.0004
Matsuda index at Baseline	4.77 (3.64–7.1)	2.47 (1.79–3.4)	0.008
HOMA-IR score at follow-up	1.48 (1.11–1.82)	3.87 (2.42–5.06)	0.0004
HOMA-IR score at Baseline	2.24 (1.36–2.86)	3.49 (2.51–5.07)	0.04
HOMA-%β at follow-up	95.96 (75.56–138.34)	129.67 (90.31–178.16)	NS
HOMA-%β at Baseline	250.87 (169.7–346.5)	201.54 (171–298.59)	NS
HbA1c at Follow-Up (%)	5.2 (5–5.4)	5.3 (5.1–5.5)	NS
Triglycerides at Follow-Up (mg/dL)	58 (50–64)	122 (74–210)	0.0002
Triglycerides at Baseline (mg/dL)	74 (51–86)	126.5 (76–172)	0.02
LDL-c at Follow-Up (mg/dL)	103.2 (86–111.4)	111.2 (103.8–132.2)	0.04
LDL-c at Baseline (mg/dL)	91.8 (78.2–100.8)	109.7 (86.0–139.8)	NS
HDL-c at Follow-Up (mg/dL)	70 (66–85)	47.5 (42–69)	0.005
HDL-c at Baseline (mg/dL)	59 (56–72)	53 (42–66)	0.04
SHBG at Follow-Up (nmol/l)	55.93 (37.66–87.35)	38.72 (23.36–66.32)	0.08
SHBG at Baseline (nmol/l)	54.09 (38.88–61.64)	26.64 (18.40–42.90)	0.02
FAI at Follow-up	2.23 (1.36–3.90)	5.4 (2.20–9.50)	0.08
FAI at Baseline	3.87 (3.44–5.24)	9.84 (4.63–11.66)	0.003

Data are expressed as median (25–75% quartiles) or numbers (%). *p* < 0.05 was considered statistically significant. Variables were analysed by Mann–Whitney U-test. BMI, body mass index; FAI, free androgen index; HbA1c, glycated haemoglobin; HDL-c, high-density lipoprotein cholesterol; HOMA-IR, homeostasis model assessment of insulin resistance; HOMA-%β, homeostasis model assessment of β-cell dysfunction; LDL-c, low-density lipoprotein cholesterol; M-clamp value, insulin sensitivity estimated with the euglycaemic hyperinsulinaemic clamp technique; NS, non-significant; SHBG, sex hormone-binding globulin.

**Table 8:** Significant predictors of prediabetes in women with a history of PCOS.

Covariates—Parameters Stated at the Beginning of the Follow-Up	OR	95% CI for OR		*p*-Value
		Lower	Upper	
BMI (kg/m^2^)	1.17	1.02	1.34	0.02
Waist Circumference (cm)	1.09	1.02	1.16	0.01
Glucose 0’ (mg/dL)	1.20	1.03	1.39	0.02
Glucose 120’ (mg/dL)	1.07	1.02	1.13	0.008
Glucose 120’-0’ (mg/dL)	1.06	1.01	1.11	0.02
Mean Glucose (mg/dL)	1.10	1.03	1.18	0.005
Insulin 120’ (uIU/mL)	1.02	1.003	1.04	0.02
Insulin 120’-0’ (uIU/mL)	1.03	1.004	1.05	0.02
M-clamp Value (mg/kgffm/min)	0.73	0.56	0.95	0.02
Triglycerides (mg/dL)	1.02	1.004	1.05	0.02
HDL-c (mg/dL)	0.94	0.88	0.99	0.04
FAI	1.42	1.09	1.86	0.01

Univariate logistic regression. Values are ORs (with 95% CI) and reflect the associations of prediabetes risk with investigated variables. *p* < 0.05 was considered statistically significant. BMI, body mass index; CI, confidence interval; FAI, free androgen index; HDL-c, high-density lipoprotein cholesterol; M-clamp value, insulin sensitivity estimated with the euglycaemic hyperinsulinaemic clamp technique; OR, odds ratio.
